# Investigation of the Thermal Characteristics of a Novel Laser Sintering Machine for Additive Manufacturing of Continuous Carbon Fibre-Reinforced Polymer Parts

**DOI:** 10.3390/polym15163406

**Published:** 2023-08-14

**Authors:** Michael Baranowski, Felix Basalla, Florian Kößler, Jürgen Fleischer

**Affiliations:** 1 Institute of Production Science, Faculty of Mechanical Engineering, Karlsruhe Institute of Technology (KIT), Kaiserstaße 12, 76131 Karlsruhe, Germany; 2 Karlsruhe Research Factory, Karlsruhe Institute of Technology (KIT), Rintheimer Querallee 2, 76131 Karlsruhe, Germany

**Keywords:** laser sintering (LS), continuous carbon fibre-reinforced polymer parts (CCFRPs), heat-affected zone (HAZ), heat distribution, operating points

## Abstract

This paper presents the thermal analysis of a novel laser sintering machine for additive manufacturing of continuous carbon fibre-reinforced polymer parts. The core element of this machine is a fibre integration unit with a heated fibre nozzle. With the help of an additional heat source, which is mounted on the bottom side of the fibre integration unit, the temperature of the powder bed surface is kept within the sintering window of the PA12 material used in the investigations. Different heat source variants differing in shape and material were analysed experimentally concerning the heat distribution achieved within the powder bed surface using an infrared camera. Based on the best-rated variant showing the most homogeneous heat distribution, operating points for successful continuous fibre integration were experimentally identified. An aluminium plate with a closed fibre nozzle slot and symmetrical surface heating power has proven to keep the powder bed surface reliably warm. Compared to the initial state, the resulting increased uniformity of heat-affected zones created by the heated fibre nozzle HAZ was evaluated by fabricating a horseshoe part made of PA12. Furthermore, a CCFRP flat pedal for mountain bikes demonstrated roving integration’s process reliability and reproducibility.

## 1. Introduction

The utilisation of Continuous Carbon Fibre-Reinforced Polymer (CCFRP) parts in industrial applications holds immense potential for effectively and economically reducing future products’ consumption and CO_2_ emissions [1]. CCFRP parts are known for their high mechanical tensile properties along the fibre direction and low weight-to-strength ratio [2].

Additive manufacturing processes offer a promising approach to producing CCFRP parts without the need for tools, allowing for efficient production with high customisation and complex shapes. Material extrusion (MEX), including fused layer modelling (FLM) and ARBURG plastic free-forming (APF), has been extensively studied for the additive manufacturing of CCFRP parts [3,4,5,6,7,8,9,10]. Another category of processes for CCFRP parts is vat photopolymerisation (VPP) [11,12,13]. However, parts produced by these processes (MEX, VPP) do not fully meet the quality requirements for the matrix. The nature of these processes necessitates using support structures that must be removed and disposed of after production, resulting in additional time and cost for disposal and post-processing steps.

Moreover, the use of support structures restricts the creation of overhangs, cavities, and undercuts, limiting the complexity of the parts. Removing support structures can also lead to surface defects on the remaining part surfaces, resulting in an inconsistent appearance. Additionally, MEX and VPP are not suitable for economical small-batch production.

In contrast, the laser sintering (LS) process offers a promising alternative for manufacturing CCFRP parts. A comparison of mechanical and thermal properties and the long-term stability of polymer parts produced through MEX, VPP, and LS demonstrate the significant advantages of the LS process. LS enables creating robust functional parts comparable to those achieved through injection moulding [14,15,16]. Laser-sintered specimens exhibit a higher Young’s modulus than injection-moulded specimens due to increased crystallinity in semi-crystalline thermoplastics. Tensile strength is nearly identical to that of injection-moulded specimens. The LS process eliminates the need for support structures, providing greater design freedom and eliminating the need for time-consuming and costly post-processing steps [16]. Furthermore, LS enables high-complexity production of near-net-shape functional parts by incorporating undercuts, cavities, and overhangs in a single process step. The LS process allows economical small-batch production by compactly positioning parts vertically and horizontally in the powder bed [14,17]. Compared to FLM parts, LS parts exhibit up to three times less anisotropy, higher dimensional accuracy, and reduced surface roughness [14,17,18]. Therefore, the LS process yields polymer parts with promising basic properties (matrix) for CCFRP parts. However, currently, no commercially available LS machines combine the advantages of the LS process with continuous fibres. Challenges in integrating continuous fibres into the LS process arise due to complex temperature control and the repetitive application movement of the recoater for each layer.

To combine the process-specific advantages of the LS process and the promising part properties with the benefits of continuous carbon fibres, the technical feasibility, i.e., the layer-related integration of continuous 1 K rovings (fibre strand with 1000 single fibres) out of carbon into laser-sintered parts made of PA12, was demonstrated by a prototypical LS machine in [19,20]. The key component of this prototype LS machine is a fibre integration unit that integrates the rovings into the already manufactured layers of the part. A heated fibre nozzle is used to liquefy the polymer locally, creating a heat-affected zone (HAZ) with specific width $b_{HAZ}$ [mm] and depth $t_{HAZ}$ [mm]. The roving is then placed into the liquefied melt through a synchronised sequence of motions between the roving feed rate and the nozzle feed rate v_D_. In [19,20], the influence of the fibre nozzle parameters and material parameters on the shape of the HAZ was investigated using a simplified experimental setup. The results from [19,20], therefore, provide the first starting points for successful fibre integration and form the basis for this paper.

However, a significant challenge for integrating rovings in the LS process represents the complex temperature control. During roving integration, the structure of the fibre integration unit causes shading of the radiation of the infrared (IR) emitters, which are responsible for a stable and homogeneous tempering of the powder bed surface, especially the part within the “Sintering Window”. Due to this shading effect, measuring and controlling the part and powder bed surface temperature is impossible with the installed IR emitters and the pyrometer. The result of that shading is an uncontrolled cooling of the part and, thus, a limited period $\tau_{HM3}$ [s] for roving integration. Due to the cooling of the part, only a roving with a length of 60 mm and a feed rate of the fibre nozzle of v_D_ = 80 mm/min could be integrated into the part. Thus, only a very low fibre volume content could be set, and, as a result, a small increase in the mechanical properties was observed [19]. Cooling of the part leads to an uneven profile of HAZ’s width and depth, which means that if the part cools down too much, the rovings can no longer be integrated deep enough into the part. The consequence is a dragging effect by the recoater and, in the worst case, the abortion of the entire printing process. Furthermore, the risk of in-build curling of the part increases as the crystallisation temperature is reached. In-build curling also results in interfering contours for the recoater. To integrate rovings deeply enough into the part and avoid in-build curling, keeping the part and the powder bed surface constant and stably warm within the sintering window during the roving integration step is crucial for successful roving integration in the LS machine developed. An additional heat source on the bottom of the fibre integration unit is intended to keep the part/powder bed surface temperature constant and homogeneous within the sintering window. This additional heat source has an adjustable temperature $T_{HM3}$ [°C] and an adjustable air gap width $h_{HM3}$ [mm] and is arranged parallel to the powder bed surface (printing plane). To keep the part and the powder bed surface within the sintering window, a comprehensive understanding of the heat flows during roving integration in the developed LS machine is indispensable to achieve a process-safe and economical production of CCFRP parts with high fibre volume content and, therefore, high mechanical properties of the parts.

This paper analyses three heat source variants concerning the achievable temperature homogeneity on the powder bed surface and operating points for a reliable and reproducible roving integration within the sintering window of polyamide 12 (PA12). Section 2.1 introduces the material used in this study and defines the associated sintering window as the basis for processing semi-crystalline PA12 in the LS machine developed. Section 2.2 describes the principle of roving integration, including the heat fluxes with heat sources and heat sinks during roving integration. Fourier’s law is used in this context, a particular case of free convection with internal flow. Finally, a summary of the influencing and target variables investigated in this study for process-reliable heat retention of the powder bed surface, particularly the part, is given in the same section. In Study 1 in Section 2.3, three heat source variants, which differ in shape and material, are analysed regarding the achievable temperature distribution on the powder bed surface. The experimental setup, the measurement setup with an infrared camera, and the investigated influencing and target variables are introduced in the same section. Based on the validity criteria of Fourier’s law, the temperature homogeneity on the powder bed surface is evaluated, and the most promising variant with the most homogenous temperature distribution is selected. In Study 2, the experimental setup to identify operating points for constant and reliable heat retention within the sintering window of the part/powder bed surface temperature is defined in Section 2.4. To verify the best-evaluated heat source (Section 2.3) and the identified operating points (Section 2.4), Section 2.5 presents the experimental setup for the fabrication of a horseshoe part out of PA12 with embedded 1K roving and the subsequent analysis of the uniformity of the generated HAZ. Furthermore, the process reliability of the achievable heat distribution and the operating points are evaluated based on a continuous fibre-reinforced flat pedal for mountain bikes. Section 3 presents and discusses the results concerning the initial state.

In this paper, an aluminium plate with a covered fibre nozzle slot (feeler gauge tape) and glued silicon heating mat with symmetrical surface heat power proved to be most beneficial to keep the powder bed surface within the sintering window during roving integration. Regarding temperature, reliable operation favoured 175 °C < $T_{HM3}$ ≤ 185 °C for an air gap width of $h_{HM3}=$ 1 mm and $T_{HM3}$ > 185 °C for an air gap width of $h_{HM3}$ = 1.5 mm for an almost constant part and power bed surface temperature within the sintering window. Compared to the initial state, the resulting increased uniformity of a HAZ was demonstrated by fabricating a horseshoe part made of PA12. A CCFRP flat pedal demonstrated the process reliability and reproducibility of roving integration.

## 2. Materials and Methods

This section describes the principle of roving integration in the LS machine concerning the influencing and target variables for keeping the part and powder bed surface temperature within the sintering window. This paper does not provide a detailed description of the machine and the achievable part properties. This can be found in [19,20,21].

### 2.1. Sintering Window of PA12

A commercially available powder from Sintratec AG is the basis matrix material for the CCFRP parts. It is a black PA12 powder with the properties listed in Table 1.

**Figure 1:**
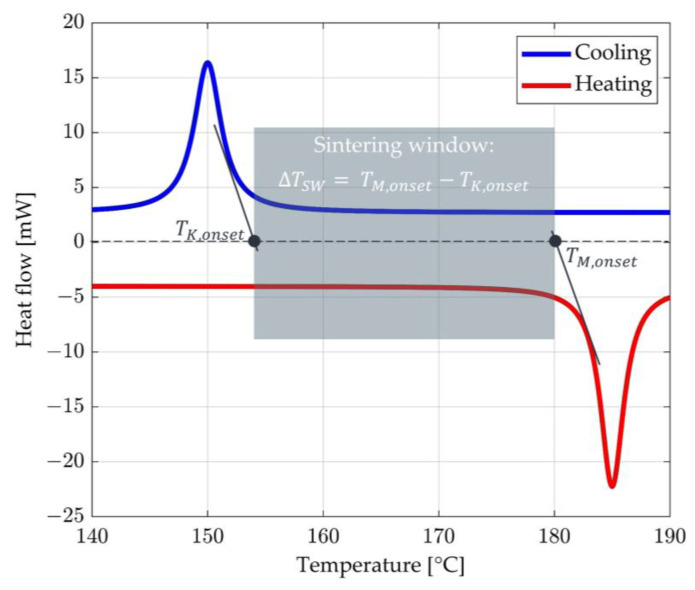
Schematic diagram of the DCS analysis for PA12 with indicated sintering window according to [14].

For semi-crystalline thermoplastics (e.g., PA12) to be processed in the LS process, they must be continuously tempered in the sintering window during the layer-wise build-up process [23,24,25,26,27]. It is located between the onset of crystallisation $T_{K,onset}$ and the onset of melting $T_{M,onset}$ and can be determined with the help of DSC analyses [14,27]. The result of a DSC analysis for PA12 is shown schematically in Figure 1 [14].

The x-axis shows the temperature in degrees Celsius, and the y-axis shows the measured heat flux in milliwatts (mW). Characteristics are the two peaks for cooling (blue) and heating (red) of the PA12. $T_{M,onset}$ describes the beginning of melting, and during cooling, $T_{K,onset}$ represents the beginning of crystallisation. Between $T_{K,onset}$ and $T_{M,onset}$ is the metastable two-phase range, the so-called “Sintering Window”. Within this window, the polymer used can be processed in the LS process, particularly for roving integration.

Section 2.2 explains the principle of roving integration with influencing and target variables for successfully keeping the part and powder surface temperature within the sintering window.

### 2.2. Principle of Roving Integration of the Developed LS Machine

**Figure 2:**
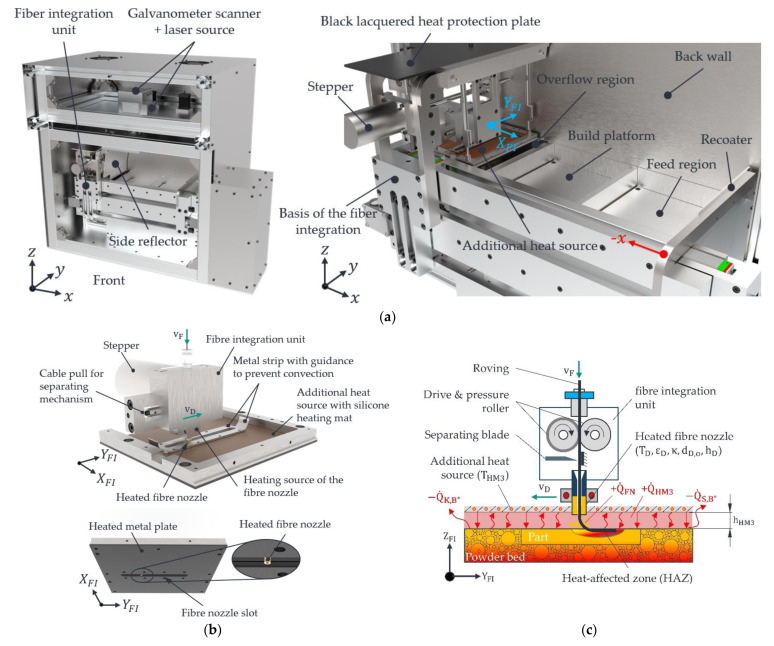
View the process chamber of the developed LS machine (**a**) with a detailed view of the fibre integration unit (**b**). Schematic representation of the heat fluxes and influencing factors (**c**).

The starting point for roving integration is a heated process chamber of the LS machine at around 110 °C. After the fresh powder has been applied by the recoater and the surface temperature of the powder bed has been homogenised by the IR emitters, the applied powder layer is melted by the laser beam. According to an ISO 6983 G-code, the layer-related (2D) integration of one or more rovings occurs sequentially. For this, the entire structure of the fibre integration unit with an additional heat source and heated fibre nozzle is moved in rapid traverse in the x and y directions to the starting point of the first roving path in the stable sintering area. The developed LS machine with a view into the process chamber is shown in Figure 2a. All symbols, incl. units, are described in Table 2 and Table 3.

The fibre integration unit and its components are shown in detail in Figure 2b,c. A metal plate with a glued silicone heating mat (additional heat source) heated to the temperature $T_{HM3}$ and arranged parallel to the powder surface at a distance $h_{HM3}$ on the bottom side of the fibre integration unit is used to keep the part and the powder bed surface within the sintering window. Heat transfer between the heated fibre nozzle at temperature $T_{FN}$ and the part surface creates a local melt zone or HAZ with a shape describing width $b_{HAZ}$ and depth $\tau_{HAZ}$ in the already melted layers of the part. At this point, the viscosity level of the polymer is locally reduced. Simultaneously with the melting process, the roving is fed through the heated fibre nozzle by a drive and pressure roller. The roving is bent between the face of the ring-shaped fibre nozzle and the generated HAZ, synchronised with the feed rate $\;v_{D}$ of the nozzle. Due to its intrinsic heat and stiffness (influenced by a coating), the roving is immersed in the liquefied polymer and, thus, in the HAZ. The coating used ensures, on the one hand, that the inherent stiffness of the roving is increased and that the roving can be fed reliably to the part within the fibre integration unit. This coating prevents kinking or canting and, thus, clogs the fibre nozzle within the fibre integration unit. On the other hand, this coating favours the adhesion of the matrix (PA12) [28]. The resulting melt wets the roving and fixes it to the underlying layers. A cutting blade above the fibre nozzle cuts the continuous roving to a length programmed in the G-code. The built-in diode laser (450 nm, 1.6 W) is inactive during roving integration. Once the rovings have been successfully integrated, the fibre integration unit moves to its home position, and the recoater applies a new layer of fresh powder. After the IR emitters have heated and homogenised the powder bed surface to the sintering temperature of around 175 °C, the laser melts the new powder layer, fully embedding the roving in the polymer matrix. This process is repeated until all rovings are integrated according to the G-code. After completion of the printing process, the powder bed with the CCFRP parts contained therein is cooled down in a controlled manner.

#### 2.2.1. Heat Fluxes during Roving Integration

To compensate for the loss of heat fluxes due to the IR emitters, an additional heat source at the bottom side of the fibre integration unit is used (see Figure 2b,c). A heat flux equilibrium is required during roving integration to keep the powder bed’s temperature, especially the already manufactured part structures, stable and constant within the sintering window of the PA12 used. This means that all heat sources and heat sinks must keep each other in equilibrium. Equation (1) below shows the total heat flux ${\dot{Q}}_{FI}$ with positive and negative heat fluxes for the period of roving integration. All symbols, incl. units, are described in Table 2 and Table 3.

(1) $${\dot{Q}}_{FI}={\dot{Q}}_{HM3}+{\dot{Q}}_{FN}{+\dot{Q}}_{Pl}-{\dot{Q}}_{K,B}-{\dot{Q}}_{S,B}-{\dot{Q}}_{M}$$

${\dot{Q}}_{HM3}$ is the heat flux introduced by the additional heat source. To create the required HAZ for embedding rovings, a heat flow ${\dot{Q}}_{FN}$ is introduced locally into the manufactured part layers with the help of the heated fibre nozzle. Since ${\dot{Q}}_{FN}$ is used solely for forming the HAZ and not for keeping the part/powder bed surface warm, ${\dot{Q}}_{FN}$ does not contribute to the heat flux equilibrium. Thus, ${\dot{Q}}_{FN}$ is not a subject of investigation in this paper. A detailed analysis of ${\dot{Q}}_{FN}$ will be carried out in [20,29]. ${\dot{Q}}_{Pl}$ describes the heat flow introduced by the platform heaters at the bottom of the building platform. However, this heat flux becomes ineffective on the part/powder bed surface as the part height or the powder bed thickness increases [30,31]. ${\dot{Q}}_{Pl}$ is, therefore, also neglected in this consideration. According to [24,32,33,34], the negative summands ${\dot{Q}}_{K,B}$, ${\dot{Q}}_{S,B}$, and ${\dot{Q}}_{M}$ describe heat losses to the environment. According to [27,34], a significant portion of the heat already introduced into the powder bed is dissipated to the atmosphere of the process chamber of the LS machine due to convection ${\dot{Q}}_{K,B}$ and radiation ${\dot{Q}}_{S,B}$. Furthermore, according to [32,34], on the mantle walls of the build chamber, the stored heat ${\dot{Q}}_{M}$ in the powder bed is emitted to the machine structure. ${\dot{Q}}_{M}$ can be compensated with the help of additional wall heaters, but they are not included in the developed LS machine. This results in a reduced 105 × 105 mm^2^ stable sintering area in the developed LS machine.

To create a heat flux equilibrium within the stable sintering area during roving integration, the additional heat source ${\dot{Q}}_{HM3}$ must be designed according to Equation (1), so that, as far as possible, all losses within the stable sintering area are compensated by the additional heat source.

#### 2.2.2. Influencing Variables for Keeping the Part and Powder Bed Surface Temperature within the Sintering Window during Roving Integration

The heat flux ${\dot{Q}}_{HM3}$ to be applied is shown in Equation (2) below. Since this work considers direct heat transport from the additional heat source to the powder bed surface, one-dimensional heat flows are considered for simplification. All influencing variables contained in Equation (2) and caused by the additional heat source are summarised and explained in Table 2. During roving integration,$T_{O}$ is a target variable, which must be kept within the sintering window (see Section 2.2.3). All symbols, incl. units, are described in Table 2 and Table 3.

(2) $${\dot{Q}}_{HM3}=\frac{\sigma }{\frac{1}{\varepsilon_{HM3}}+\frac{1}{\varepsilon_{P}}-1}\cdot A_{HM3}\cdot {(T}_{HM3}^{4}-T_{O}^{4})+\lambda_{L}\cdot A_{HM3}\cdot \frac{T_{HM3}-T_{O}}{h_{HM3}}$$

The first summand represents the Stefan–Boltzmann law, with an additional radiation exchange between the additional heat source (black lacquered metal plate) and the powder bed surface. The second summand in Equation (2) represents Fourier’s law, a particular case of free convection with internal flow within plane horizontal plates. A derivation and detailed description of the physical laws of free convection with internal flow will not be given here. For this, reference is made to [35]. However, the following validity criteria must be fulfilled during the roving integration, according to [35,36], for Fourier’s law to be applied in the developed LS machine.

- $T_{HM3}$ of the additional heat source must be hotter than the powder/part surface temperature $T_{O}$ ($T_{HM3}>$ $T_{O}$). In this case, the Rayleigh number Ra is <1, the fluid is stably layered, and no flow is generated (Nusselt number Nu = 1).
- Large aspect ratio between the shortest length $b_{HM3}$ of the additional heat source and the air gap width $h_{HM3},$ according to the following Equation (3).
  (3) $$\Gamma =\frac{b_{HM3}}{h_{HM3}}\gg 1$$
- Adiabatic sidewalls at the edges of the additional heat source to avoid convection flows and, thus, cooling the powder bed surface at the edge areas.
- Closed top (metal plate of the additional heat source) and bottom (powder bed) layer, so that the occurrence of convection within the tempered area is avoided.

The first condition $T_{HM3}>T_{O}$ can be considered satisfied with the help of a glued silicone heating mat on the black lacquered metal plate with a PID controller and the fact that the temperature of the silicone heating mat can be set higher than the melting temperature of the PA12 used. The second condition can also be fulfilled according to Table 2 since the shortest side of the additional heat source $b_{HM3}$ can be set much larger than the air gap width $h_{HM3}$. The third condition is initially assumed to be fulfilled since the surface area of the additional heat source (metal plate) can be set larger than the stable sintering area of 105 × 105 mm^2^. This assumption is checked in Study 1. The last criterion is the need for closed bottom layers and top layers [35]. A closed powder layer as the bottom layer can be assumed to be a fulfilled pre-condition, since fresh powder is successively applied equally on the built platform. However, challenges arise for the metal plate from being heated by the silicone heating mat defined as the top layer. According to Figure 2b, a fibre nozzle slot in the heated metal plate is essential for the fibre nozzle to move along the y-axis. This nozzle slot violates the last validity criteria. The introduced heat $T_{HM3}$ and the stored heat of the powder bed surface $T_{O}$ are lost due to the onset of convection flows through the nozzle slot. To meet the requirement for a closed top layer, a heat source variant with a closed nozzle slot is introduced in Section 2.3. The validity criteria for Fourier’s law and the variables listed in Equation (2) summarise the influencing variables for keeping the part/powder bed surface temperature within the sintering window during roving integration in the developed LS machine. The influencing variables are listed in Table 2. This analysis does not consider influences due to the LS process (“laser-part interactions”, material composition, the influence of the heat flux of the fibre nozzle on the HAZ, part warpage due to roving integration, and ageing effects of the powder). They are kept constant as far as possible in the studies—see Section 2.3 and Section 2.4.

Initial studies were carried out in [19,20] for the influencing variables listed in Table 2. Furthermore, operating points were identified, at which 1 K rovings can be integrated into the part [19,20]. These operating points form the starting point for the first study described in Section 2.2.

#### 2.2.3. Target Variables for Keeping the Part/Powder Bed Surface Temperature within the Sintering Window

Table 3 lists the target variables addressed in this paper and their units, which are responsible for keeping the part and powder bed surface temperature warm. The overall objective of these target variables is to create the necessary prerequisite for process-reliable and reproducible roving integration.

**Figure 3:**
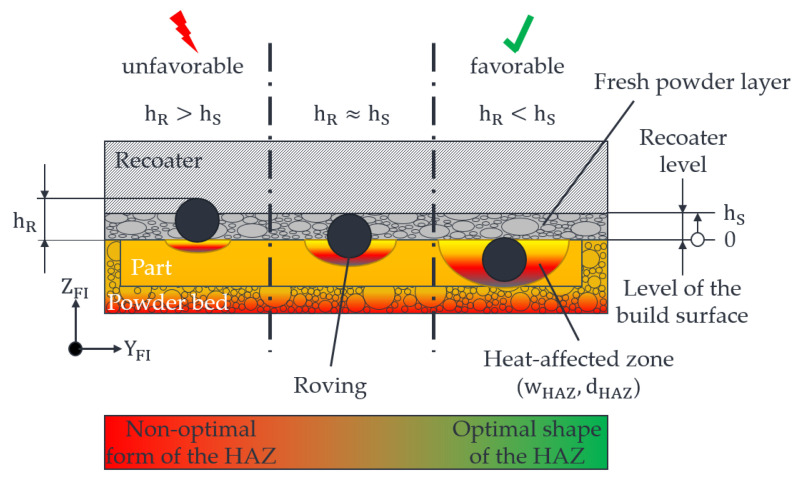
Schematic representation of the influence of the shape of the HAZ on the integration depth of a roving in the HAZ. Here, $h_{R}$ describes the roving protrusion from the part surface and $h_{S}$ the layer thickness of the LS process.

As described in Section 1, inadequate heating of the part and powder bed surface during roving integration results in significant temperature differences between the heated fibre nozzle and the fluctuating values for $T_{O}$ on the powder bed surface. At points with high temperature differences, the fibre nozzle has to apply more thermal energy to melt the polymer compared to the points with low temperature differences—see Figure 3.

In the worst case, the roving cannot be embedded deep enough into the part, which leads to an entrainment effect by the recoater in the subsequent recoating process. Therefore, the aim is to create as constant a heat distribution as possible, with ideally ∆T = 0 °C on the powder bed surface, and, thus, to obtain as constant values as possible for $b_{HAZ}$ and $t_{HAZ}$.

Furthermore, to maximise the fibre volume content (number of rovings) of the parts and, therefore, the mechanical properties in future investigations, $T_{O}$ must be kept in the sintering window as constant as possible. The period when the sintering window can be kept stable during roving integration is defined as $\tau_{HM3}$.

In Study 1 in Section 2.3, three heat source variants are analysed and evaluated regarding the homogeneity of heat distribution ∆T and the degree of fulfilment of the validity criteria for Fourier’s law. The best-evaluated variant serves in Study 2 as the basis for determining optimal operating points with a maximum value for $\tau_{HM3}$ with constant temperature $T_{O}$.

### 2.3. Study 1: Analysis and Evaluation of Three Heat Source Variants Concerning Heat Distribution

The aim of Study 1 is to analyse and evaluate three heat source variants concerning the homogeneity of the heat distribution with the lowest possible temperature differences on the powder bed surface. Furthermore, each heat source variant is to be assessed regarding the validity criteria for Fourier’s law.

#### 2.3.1. Experimental Design

Based on the validity criteria for Fourier’s law, this section presents three different heat source variants of the additional heat source, consisting of a silicone heating mat and a black lacquered metal plate. The test sequence with the measurement chain with an IR camera and the machine settings are given in Section 2.3.2 and Section 2.3.3. The heat distribution of the LS process without roving integration in will be used as a reference for the comparison.

**Figure 4:**
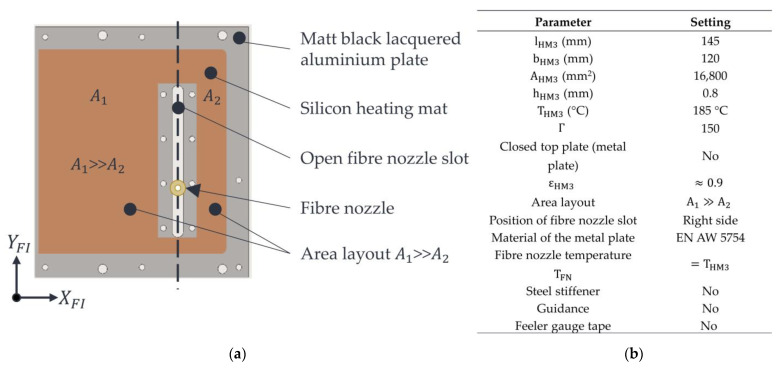
Illustration of variant 1 (**a**) with settings (**b**).

Variant 1 is shown in Figure 4a. The properties of the variant and the machine settings used are shown in Figure 4b. Variant 1 consists of a silicone heating mat manufactured (HKE-tec GmbH & Co. KG, Pfarrkirchen, Germany) with an asymmetrical surface area layout $A_{1}\gg A_{2}$. This silicone heating mat is bonded to the aluminium (EN AW 5754) metal plate that needs to be heated by a silicone adhesive Dow Corning^®^ 736 Heat Resistant Sealant (Dow Chemical Inc., Midland, TX, USA). The nozzle slot has a width of 6 mm and a length of approx. 80 mm. Due to the presence of an open nozzle slot, the occurrence of convection and local cooling of the powder bed surface in the area of the nozzle slot is to be expected. Furthermore, the inhomogeneous temperature distribution within the stable build area due to the asymmetric surface distribution and the silicone heating mat’s associated inhomogeneous surface heating performance is suspected. Further challenges could arise from the thermal expansion of the aluminium plate at high temperatures. If the expansion is too high, the installed plate may form a bulging shape. This bulging would cause the plate to touch the powder bed, thus damaging the powder bed surface or the part itself. If the temperature gets too high, powder particles could also adhere to the metal plate’s lacquered surface, and additional interfering contours could arise. These interfering contours represent additional errors and affect the process reliability of the roving integration. At worst, the manufactured parts would be pulled along by these adhesions, leading to an abortion of the printing process.

**Figure 5:**
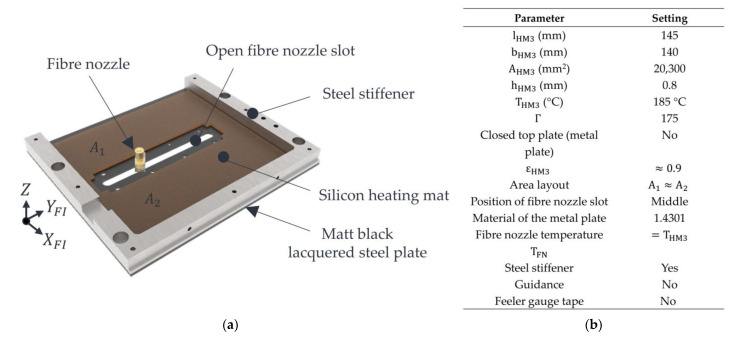
Illustration of variant 2 (**a**) with settings (**b**).

Variant 2 is shown in Figure 5a. The properties of this variant and the machine settings used are shown in Figure 5b.

Variant 2 has an almost central arrangement of the nozzle slot and, thus, a silicone heating mat with a more uniform surface heating performance. To limit the thermal expansion of the metal plate, stainless steel (1.4301 according to DIN 17007 or AISI 304) is used as material for the metal plate with an additional steel stiffener against thermal expansion. However, due to the thermal conductivity of stainless steel being up to ten times lower than that of the aluminium alloy selected in variant 1, a more inhomogeneous heat propagation in the metal plate is expected. Furthermore, the nozzle slot in this variant is open in the same way as in variant 1, so that the requirements for Fourier’s law are not entirely fulfilled, and convection flows are to be assumed.

**Figure 6:**
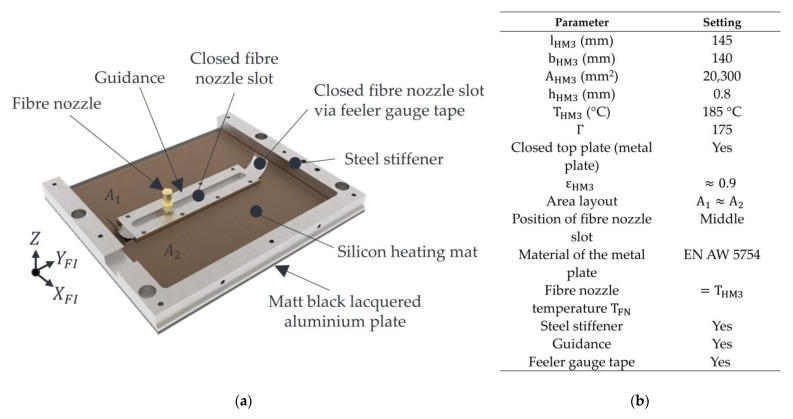
Illustration of variant 3 (**a**) with settings (**b**).

Variant 3 is shown in Figure 6a. The properties of this variant and the machine settings used are shown in Figure 6b.

A feeler gauge tape (Hasberg Schneider GmbH, Bernau am Chiemsee, Germany) with a thickness of 0.05 mm and a width of 12.7 mm in variant 3 is intended to prevent convection flows from occurring through the nozzle slot. This piece of feeler gauge tape, about 250 mm long, is guided through a guidance and, thus, covers the nozzle slot. In addition, this piece of steel foil has a centrally arranged hole serving as a passage for the fibre nozzle. The diameter of the hole corresponds to the nozzle diameter of the fibre nozzle, so that the convection that would be expected in this area is nearly avoided. When the fibre nozzle is moved along the y-axis, the installed feeler gauge tape is moved along and guided through the hold-down element (Guidance). This arrangement is intended to avoid the onset of convection over the entire nozzle slot, thus fulfilling the condition for closed parallel top layers. An inhomogeneity in the temperature distribution of the powder bed surface in the area of the nozzle slot is to be expected due to the areas that cannot be heated by the silicone heating mat and the steel stiffener. Furthermore, the metal plate in this variant is made of aluminium (EN AW 5754). To reduce the deformation of the aluminium plate and, thus, reduce the risk of bulging and adhesion of polymer powder to the plate, the same stiffening is used as in variant 2.

#### 2.3.2. Measurement Set-Up with IR Camera

**Figure 7:**
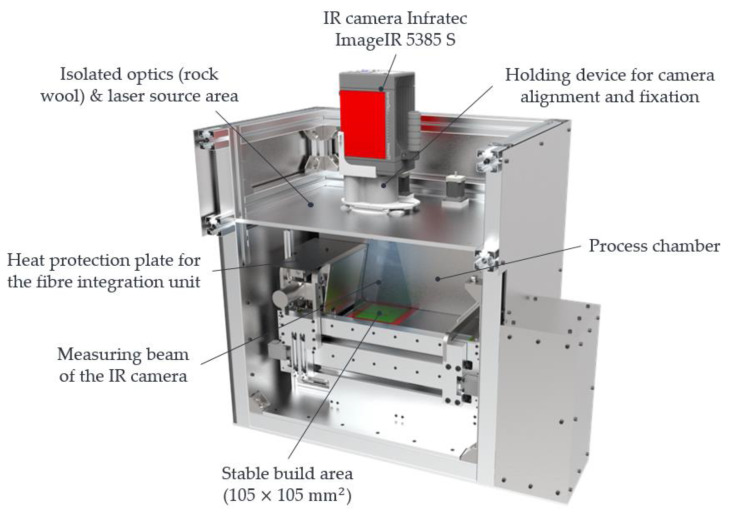
LS machine with IR camera.

Due to the poor accessibility of sensors inside the fibre integration unit or between the heated metal plate and the powder bed surface, an IR camera is used to measure the temperature distribution. The measurement setup with the LS machine and IR camera is illustrated in Figure 7 and is based on [32,33].

The data of the measuring chain with an IR camera can be taken from Table 4.

To ensure that thermal energy can be transferred to the powder bed through the heated metal plate, the fibre integration unit is positioned in the centre over the powder bed surface in rapid traverse. The measurement beam of the IR camera and the IR radiation is cut off at that instant by the fibre integration unit. Due to this shadowing, the surface temperature of a black lacquered heat protection plate on the top side of the fibre integration unit is measured by the pyrometer instead of the powder bed surface temperature (see Figure 2a). The IR emitters and the pyrometer control a reduced temperature of 165 °C on the heat protection plate. The fibre integration unit remains above the powder bed according to a specified dwell time $\tau_{HM3}$, which depends on the time required for a roving integration (roving length, number of rovings per layer). During this dwell time $\tau_{HM3}$, heat is transferred between the heated metal plate and the powder bed surface. After this dwell time $\tau_{HM3}$, the fibre integration unit moves at its home position in rapid traverse. When a threshold value of the x-axis of the fibre integration unit is reached, the temperature control returns to the sintering temperature of 175 °C on the powder bed surface with the help of the IR emitters and the pyrometer. During the entire time, and especially when the powder bed surface becomes visible to the IR camera again, continuous recording of image data takes place with the help of the IR camera. This process enables a specific measurement of the temperature distribution of the powder bed surface, which was generated by the heated metal plate. Falsifying effects caused by the IR emitters during the heating process are largely reduced by selecting the maximum possible traverse speed and, thus, the fastest possible release of the measuring field in combination with continuous image data recording by the IR camera.

#### 2.3.3. Experimental Procedure and Evaluation

The three variants will be investigated and evaluated using the measurement chain presented in Section 2.3.2 and the machine and material settings listed in Table 5.

**Figure 8:**
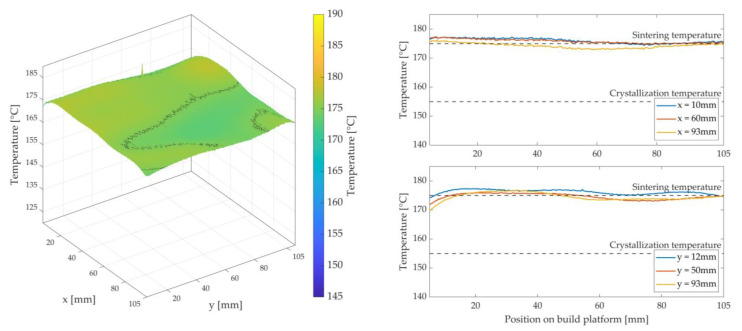
Heat distribution as a 3D plot (**left**) during the layer-wise build-up process of the pure LS process, i.e., without roving integration. Temperature profile as a 2D plot (**right**) for selected positions in the stable sintering area with the specification of the sintering window.

The LS machine is started up for each test to obtain the most accurate information possible for the developed LS machine, as if a normal sintering process would occur. After the machine has been heated, fresh powder is repeatedly applied by the recoater at sintering temperature. Subsequently, the fibre integration unit moves centrally above the built platform in rapid traverse to keep the stable build area warm for a defined dwell time $\tau_{HM3}$, ideally without interruption by the installed heat sources. In Study 1, the dwell time $\tau_{HM3}$ is defined in advance and describes the time it takes approximately to insert a roving with a length of 60 mm by a fibre nozzle feed rate $v_{D}$ = 120 mm/min. The image data obtained when moving away the fibre integration unit will be evaluated. The results for the temperature distribution are illustrated and evaluated in MATLAB as 3D plots—see Figure 8.

Statements on the degree of fulfilment of the validity criteria regarding Fourier’s law and on the temperature homogeneity are made using a graphical comparison of the generated 3D plots. The result will be a variant that shows the best possible temperature distribution and the highest degree of fulfilment of the criteria from Section 2.2.2. In addition, possible optimisation approaches for better homogeneity will be discussed.

### 2.4. Study 2: Identification of Operating Points for Process-Reliable and Reproducible Roving Integration

The heat source variant with the most homogeneous heat distribution and the best possible degree of fulfilment of the validity criteria for Fourier’s law serves as the basis for Study 2. The aim of Study 2 is to identify operating points for stable and reproducible roving integration. As described in Section 2.2.3, for parts with a high fibre volume content, the temperature $T_{O}$ must be kept as long as possible within the sintering window, so the rovings can be integrated reliably and process-safely.

#### 2.4.1. Experimental Design

According to Fourier’s law, the air gap distance $h_{HM3}$, the additional heat source temperature $T_{HM3}$, and the surface areas involved in the heat conduction form essential influencing variables. According to the Stefan–Boltzmann law, the emissivities of the powder and the lacquered metal plate are further influence variables [35]. The surface area of the powder bed, the heated metal plate, and the emissivities cannot be changed during the entire printing process and are, therefore, to be regarded as constant in this consideration. Therefore, the aim is to investigate and quantify the influence of the temperature of the heated metal plate $T_{HM3}$ of the additional heat source and the air gap distance $h_{HM3}$ on the $T_{o}\left(\tau_{HM3}\right)$ relationship. The factor levels used in this investigation are listed in Table 6**.**

#### 2.4.2. Experimental Procedure and Evaluation

The experimental procedure is based on the procedure described in Section 2.3.2 and Section 2.3.3. Differences occur in the variable dwell time $\tau_{HM3}$ of the fibre integration unit above the powder bed surface and at the measuring point for $T_{HM3}$. Considering this, not the entire powder bed surface of the stable build area is measured as described in Section 2.3.2, but a measuring point in the centre of the powder bed surface of the stable build area. Based on Fourier’s law, it is assumed that premature melting can be observed on the powder bed surface at low air gap widths and high temperature settings of the silicone heating mat (extreme case 1). In contrast, it is assumed that if the air gap width is too high and the temperature setting of the silicone heating mat is too low, there is a risk of cooling, i.e., the polymer temperature is falling below the crystallisation temperature (extreme case 2). These effects are to be analysed within the scope of this investigation. As a result, reliable operating points for the air gap width $h_{HM3}$ and the temperature $T_{HM3}$ of the installed silicone heating mat should be available within these extreme cases. The measurement results of the IR camera are evaluated using MATLAB.

### 2.5. Verification of the Heat Source Variant and the Operating Points

To verify the best-evaluated heat source variant from Study 1 and the identified operating points from Study 2, the production of two demonstrators with embedded 1 K rovings is carried out in this section.

#### 2.5.1. Evaluation of HAZ and Using a Horseshoe Part with Integrated 1 K Roving

**Figure 9:**
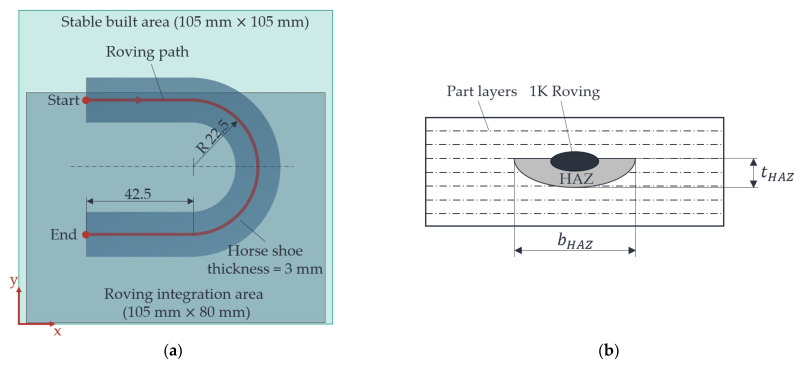
Two-dimensional representation of the horseshoe part with roving (red line) within the stable sintering area (**a**), as well as exemplary representation for measuring the HAZ (**b**).

In this section, the production of four horseshoe parts with a 1 K roving is carried out to evaluate the uniformity of the HAZ between the beginning and the end of the roving integration. A horseshoe part with inserted roving is used as a simplified part to achieve a high dwell time $\tau_{HM3}$ of the fibre integration unit over the powder bed surface, depending on the fibre nozzle feed $v_{D}$. Based on the results of the HAZs, the initial state of this paper, i.e., without an additional heat source, is to be compared with the process understanding elaborated in this paper, i.e., with the best-evaluated heat source variant and identified operating points. The same part without the influence of the additional heat source, i.e., with the risk of cooling the part/powder bed surface temperature, is used as a reference. Thus, the initial state (without additional heat source) is to be compared with the findings in this paper. The horseshoe part with the roving path indicated (red line) is shown in Figure 9 below.

The machine settings and the 1 K roving used to produce the horseshoe part are listed in the following Table 7. The material, the mixing ratio, and the other machine settings can be found in Table 5.

The HAZs are prepared according to [20]. The width and depth of the HAZs are measured using a microscope (Keyence VHM 7000). With the help of a newly developed MATLAB app, the horseshoe parts and the flat pedal in Section 2.5.2 are sliced. With this app, the G-code for the roving paths can be generated automatically, and the complete LS machine can be controlled, in addition to the part slicing [37].

#### 2.5.2. Evaluation of Process Reliability Using a Flat Pedal for Mountain Bikes

**Figure 10:**
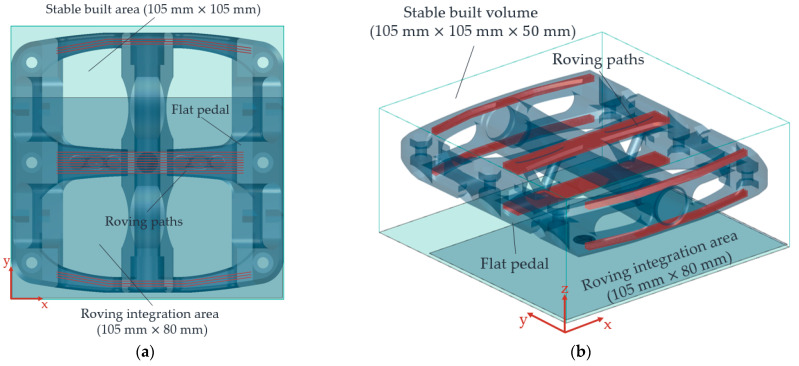
Two-dimensional representation of the flat pedal showing the individual roving paths (**a**), and a three-dimensional illustration of the flat pedal within the built volume showing the distribution of the rovings inside the flat pedal (**b**).

To test the reproducibility and consistency of the heat retention with the help of the selected heat source variant, a customised and individual flat pedal for a mountain bike is produced—see Figure 10. This flat pedal is locally reinforced with 1 K rovings at the points with increased tensile and bending stress. Each 1 K roving is shown as a single line in Figure 10a. Due to the fibre integration unit’s mechanical construction, particularly the roving extruder, the fibre area with 105 × 80 mm^2^, in which rovings can be placed in the part, is smaller than the stable sintering area. For this reason, only those rovings are integrated into the part located within the roving integration area. For illustration purposes, all possible locations for roving integration are indicated in Figure 10.

This flat pedal has a dimension of 105 × 102 mm^2^ and, thus, almost fills the stable sintering area. In particular, the process reliability of keeping the sintering window without in-build curling at the edge areas of the stable sintering area during roving integration must be checked. In addition, the number of rovings per layer and, thus, the influence of the dwell time $\tau_{HM3}$ is to be evaluated based on the identified operating points. There are up to 12 rovings in a layer. With a set nozzle feed rate of $v_{D}$ = 120 mm/min, the dwell time of the fibre integration unit is about 480 s. The same values as in Table 5 and Table 7 are manufacturing parameters. The relative horizontal roving distance (print plane) is 1 mm. The relative vertical roving spacing in the build-up direction (z) is 0.5 mm. $v_{D}$ is derived from [20,29], and the relative roving distances are from [21].

## 3. Results and Discussion

### 3.1. Heat Source Variant with the Most Homogeneous Heat Distribution

**Figure 11:**
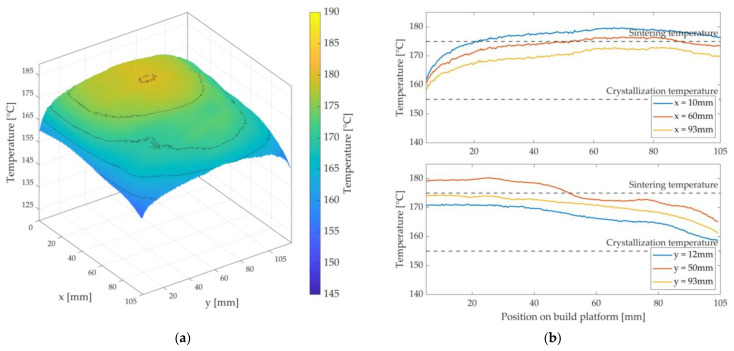
Three-dimensional representation of the measured heat distribution (**a**) and temperature curves of variant 1 at selected positions on the x- and y-axis (**b**) with the display of the sintering window.

**Figure 12:**
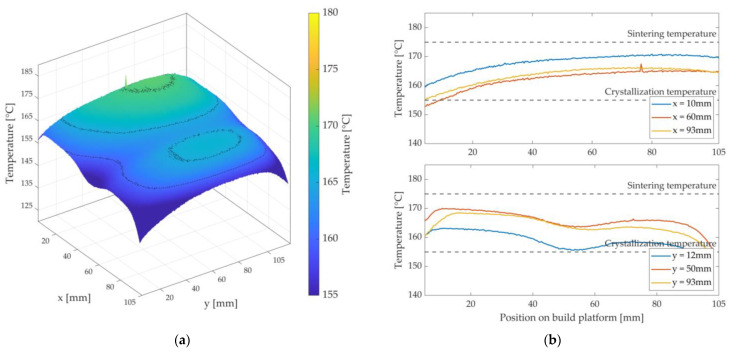
Three-dimensional representation of the measured heat distribution (**a**) and temperature curves of variant 2 at selected positions on the x- and y-axis (**b**) with the display of the sintering window.

**Figure 13:**
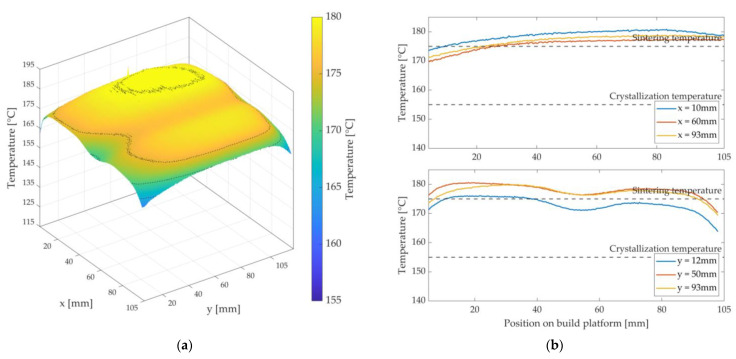
Three-dimensional representation of the measured heat distribution (**a**) and temperature curves of variant 3 at selected positions on the x- and y-axis (**b**) with the display of the sintering window.

Figure 11, Figure 12 and Figure 13 show the achieved heat distributions for variants 1–3. The x-axis and y-axis correspond to the directions of the powder bed axis. On the z-axis, the temperature values are plotted. In addition, the temperature curves for selected positions on the powder bed surface are shown along the x-axis and y-axis. First, the three variants are presented, followed by a description of the results or the characteristics of the respective variant, with a subsequent discussion.

For variant 1, a temperature decrease along the x-axis is observed—see Figure 11.

In addition, there is a temperature decrease along the y-axis in the middle of the x-axis. Furthermore, a hot spot can be seen in the rear area of the measuring field (3D plot) towards the overflow region (x = 0 mm)—see Figure 2a. A curved shape can be observed along the y-axis. A significant temperature drop can be observed at the edges and at the corners. The maximum temperature is approx. 179.5 °C, and the lowest temperature within the stable build area is 153 °C (∆T = 26.5 K).

According to Fourier’s law, the heating surface area involved is directly proportional to the heat output transferred. The asymmetric surface heating power ($A_{1}\neq A_{2}$) of the silicone heating mat leads to a higher heat flux in the half that is directed towards the overflow region than in the right area (x > 40 mm) of the nozzle slot and, thus, to a decrease of the temperature along the x-axis. The slight temperature decrease along the y-axis (50 mm < y < 75 mm) in the middle of the x-axis can be explained by an open nozzle slot. Due to this nozzle slot, the condition of a closed upper layer is violated. In this area, Nu > 1 is to be assumed, whereby the occurrence of convection flows is listed as the most probable cause. The result is a cooling of the powder bed surface. Furthermore, there is a risk of premature crystallisation at the edge, the corners, and in the right area of the nozzle slot (x-axis). The most probable causes are a cooling effect of the metal plate by the ambient atmosphere and the lack of chamber wall heaters in the prototypical LS machine. A possible countermeasure would be to increase the temperature $T_{HM3}$ of the metal plate, but at the cost of a more substantial hot spot formation in the left area of the nozzle slot. Compared to the pure LS process reference in Figure 8, the investigated variant 1 shows significant deviations. It can be said that this variant has a strongly inhomogeneous heat distribution. In addition to premature crystallisation of the parts (and thus warpage), this would result in an uneven shape of the HAZ. So, this variant insufficiently satisfies the validity criteria of Fourier’s law. Therefore, this variant is classified as unusable.

At first glance in variant 2 in Figure 12, the wavy, two-hump shape of the temperature distribution along the x-axis and over almost the entire length of the y-axis is noticeable.

Furthermore, the overall temperature level is lower than in variant 1. The temperature at the edges and corners is also lower than in variant 1. The maximum temperature is approx. 170 °C, and the minimum temperature at the edges or corners is 150 °C (∆T = 20 K).

Due to the open nozzle slot, the introduced heat of the silicone heating mat and the stored heat in the powder bed enter the environment of the process chamber by convection flows. A cooling of the surface along the nozzle slot is the result. In addition, the steel used as material for the metal plate has a thermal conductivity of up to 10 times lower than that of aluminium (variant 1) [35,38]. This means that the heat energy introduced by the silicone heating mat is not evenly distributed in the metal plate. The heat is concentrated in the centre of the two main surface areas $A_{1}\;$ and $A_{2}$. The heat energy emitted towards the edge areas of the metal plate decreases due to the comparatively cooler steel stiffener and the cooler ambient temperature. In addition, the validity criteria (Nu = 1) are increasingly violated in the edge areas. The result is an increased cooling of the powder bed surface at the corners and edge areas. To avoid this cooling, either the heating mat should emit more heat in the edge areas, the stiffener should be made thinner, and/or the metal plate should be made of aluminium. Compared to the reference for the pure LS process in Figure 8, this investigated variant 2 also shows substantial deviations. The validity criteria are rated as insufficient for this variant and, thus, variant 2 is also rated as unusable.

In variant 3 in Figure 13, an overall more homogeneous and higher temperature level can be observed at first glance. Along the x-axis, a slight decrease in temperature can be seen compared to variant 1 and variant 2. Like in the first variants, depression can be seen along the y-axis and in the middle of the x-axis. A drop in temperature can also be seen in the corner areas and at the edges, but not quite as much as in the first two variants. The temperature difference between the hot spot (approx. 178 °C) and the cold spot (approx. 165 °C) is approx. ∆T = 13 K.

It can be seen that the obtained heat distribution in this variant is very similar to the heat distribution of the pure LS process in Figure 8. The comparatively small depression in the centre of the x-axis can be attributed to the nozzle slot, which is present and sealed by the feeler gauge tape. Since the hold-down element and the feeler gauge tape are not appropriately heated by the bonded silicone heating mat, they have a lower temperature than the set temperature $T_{HM3}$ for keeping the powder bed surface warm. The result is a reduced temperature level within the built area of the hold-down element and, thus, a reduced heat flux onto the powder bed surface. However, using a feeler gauge tape reduces a sharp temperature decrease. The temperature decrease at the corners and edges can be explained by the ambient temperature and the installed and relatively cold stiffener. As in the first two variants, both the ambient atmosphere and the stiffener extract heat energy from the edge area of the metal plate, causing the latter to cool at the edges and corners. The heat flux transferred to the powder bed surface is lower at these points. In addition, the condition according to Nu = 1 is increasingly violated in the edge areas. However, if this decrease is compared with variant 1 and variant 2, a significantly smaller decrease can be observed. Overall, variant 3 fulfils the validity requirements more satisfactorily than the first two variants. In an area of approx. 105 mm × 105 mm on the built platform, these validity criteria can be considered to be fulfilled. Variant 3 is, therefore, rated as usable and forms the basis for the following considerations. 

### 3.2. Operating Points for Stable Keeping Warm of the Part and Powder Bed Surface Temperature within the Sintering Window

**Figure 14:**
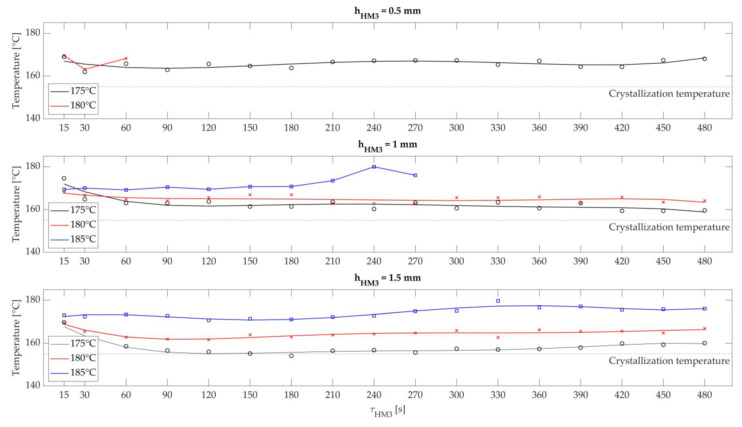
Determined temperature–time curves with three different air gap widths $h_{HM3}$.

Based on the best-rated variant from Section 3.1 (variant 3), this section analyses the influence of the air gap width $h_{HM3}$, the temperature of the metal plate $T_{HM3},$ and the dwell time $\tau_{HM3}$ of the fibre integration unit above the powder bed surface on the temperature–time $T_{HM3}\left(\tau_{HM3}\right)$ behaviour. The aim is to identify the powder bed surface’s time-dependent temperature profile and, thus, determine stable operating points for successful roving integration. The results are first presented and then discussed. Finally, possible optimisation approaches for improving the time-dependent temperature behaviour are listed. Figure 14 shows the results of this investigation.

Three temperature–time diagrams are shown for the three air gap widths investigated. The dwell time in seconds is plotted on the x-axis, and the temperatures measured with the IR camera in degrees Celsius are on the y-axis.

Air gap width of $h_{HM3}$ = 0.5 mm: The temperature curves for $T_{HM3}$ = 175 °C and $T_{HM3}$ = 180 °C can be seen, whereby the curve for $T_{HM3}$ = 180 °C stops after approx. 60 s. Furthermore, between 30 and 60 s, the temperature curves decrease to approx. 162 °C and then increase slightly to approx. 168 °C until the end of the dwell time $\tau_{HM3}$. Starting from the lowest measured value, all the temperature values determined are 7 °C above the crystallisation temperature.

During that observation, a temperature increase of the heated metal plate to over $T_{HM3}$ ≥ 180 °C resulted in premature melting of the powder bed surface. Furthermore, the initial drop in temperature of the powder bed is due to the transition between the supplied heat fluxes, i.e., the heat provided by the IR emitters to the heated metal plate ${\dot{Q}}_{HM3}$. As soon as the fibre integration unit completely covers the powder bed surface, the cooling of the surface starts because the air gap contains a comparatively colder ambient atmosphere as the heat transfer medium at the beginning. So, before warming up the powder bed surface, the air gap must be made hotter. Furthermore, some tests had to be repeated several times due to the relatively small air gap width of 0.5 mm. The reason for this was the adhesion of powder particles on the metal plate, which was not optimally flat due to thermal expansion. These adhesions led to damage on the loose powder bed surface in areas with a local, low air gap width. Although the crystallisation temperature was not reached in this experiment, the reduced air gap width would endanger the process reliability due to adhesions on the metal plate for later roving integration. An air gap width of 0.5 mm is, therefore, considered unsuitable for automated roving integration.

Air gap width of $h_{HM3}$ = 1.0 mm: Similarly to the air gap width of 0.5 mm, a decrease in the powder bed temperature can be seen initially of up to $\tau_{HM3}$ = 90 s. The temperatures were kept relatively constant over time. A slight temperature increase towards higher dwell times can be observed. Furthermore, the measured values stop at a set metal plate temperature of $T_{HM3}$ = 185 °C after approx. $\tau_{HM3}$ = 270 s. The temperature curves for a metal plate temperature are approx. 4 °C above the crystallisation temperature for $T_{HM3}$ = 175 °C and approx. 10 °C above the crystallisation temperature for $T_{HM3}$ = 180 °C. The temperature curves for a metal plate temperature of $T_{HM3}$ = 185 °C and $T_{HM3}$ = 180 °C are also slightly higher than the crystallisation temperature.

In this case, Fourier’s law can be used as a reference. By increasing the air gap width to 1 mm, the temperature difference regarding the heated metal plate must be increased for the same heat energy [35]. This leads to the need for a higher temperature of the heated metal plate to guarantee stable temperature control above the crystallisation temperature. Temperatures with $T_{HM3}$ < 175 °C show an increased risk of cooling the powder bed. Too high temperatures of $T_{HM3}$ > 185 °C cause premature melting of the powder bed surface. The stable process range in this evaluation is at 175 °C < $T_{HM3}$ ≤ 185 °C. The initial cooling of the powder bed surface for $\tau_{HM3}$ = 90 s is due to the more significant amount of air to be heated in the air gap in the same manner, as it had to be heated up for an air gap width of 0.5 mm. Powder adhesion was not observed with this air gap width. Therefore, the ranges mentioned above are usable operating points.

Air gap width of $h_{HM3}$ = 1.5 mm: With an air gap width of 1.5 mm, there is an initial decrease in the surface temperature of the powder bed for up to approx. $\tau_{HM3}$ = 150 s. Higher temperatures are required for the metal plate to maintain the powder bed surface temperature above the crystallisation temperature. At temperatures below $T_{HM3}$ ≤ 175 °C, an increased undershoot of the crystallisation temperature was observed. For $T_{HM3}$ = 180 °C, the lowest point is about 6 °C, and for $T_{HM3}$ = 185 °C, the lowest point is about 16 °C above the crystallisation temperature.

As the air gap width increases, the temperature of the metal plate to be set shifts to higher values according to Fourier’s law. More heat energy must be applied for stable temperature control above the crystallisation temperature. In addition, due to the higher air gap, colder ambient air is trapped between the heated metal plate and the powder bed surface. The duration to heat up this enclosed air volume needs more time than the air gap widths of 0.5 mm and 1.0 mm. Stable operating points were identified for this variant at $T_{HM3}$ > 185 °C. However, a subsequent analysis of the temperature distribution on the powder bed surface showed a shrinking to about 90 mm × 90 mm of the stable build area. The reason for that is the increased air gap width and, thus, the continuous reduction of the aspect ratio Γ (see validity criteria for Fourier’s law).

### 3.3. Evaluation of the Heat Source Variant and the Operating Points

#### 3.3.1. Evaluation of the HAZ Using a Horseshoe Part with Integrated 1K Roving

**Figure 15:**
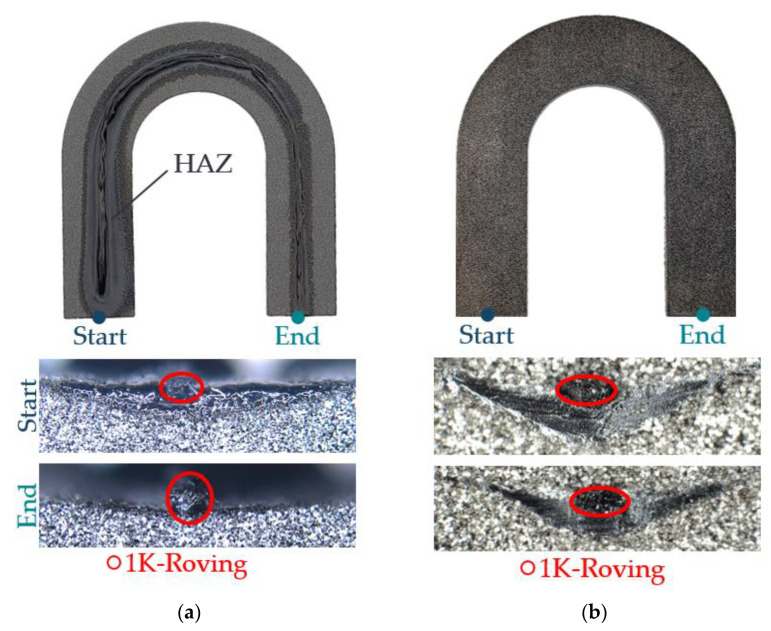
Non-uniform HAZ and different integration depths of the roving between the start and end of roving integration (**a**) for a horseshoe part without the additional heat source (initial state). More uniform HAZ due to the use of an additional heat source (**b**).

For this investigation, an air gap width of $h_{HM3}$ = 1 mm and a metal plate temperature of $T_{HM3}$ = 185 °C were selected as operating points. Figure 15 shows the generated HAZs in the horseshoe parts.

**Figure 16:**
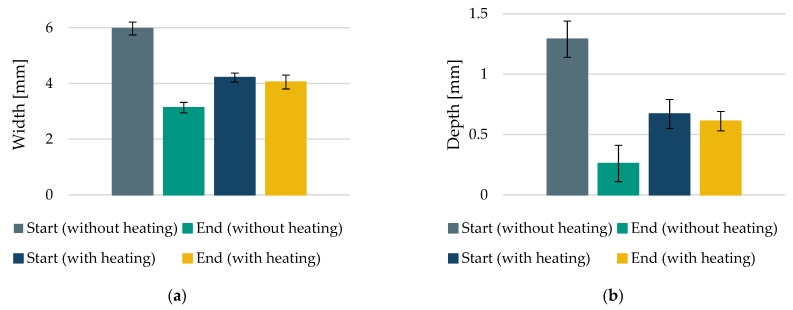
Comparison of the horseshoe part with and without additional heat source concerning width (**a**) and depth (**b**) of the generated HAZ.

On the left-hand side (Figure 15a) is the HAZ for the horseshoe part without additional heating (initial state of this work). On the right-hand side (Figure 15b) is the HAZ with the best-rated variant from Section 3.1. For the horseshoe part without an additional heat source, no additional powder layers could be applied due to the strong heightening of the roving at the end of the roving path. The process was, therefore, stopped at this point. A HAZ was generated for the horseshoe part with an additional heat source, which allowed a complete embedding below the level of the recoater. The 1K roving could, thus, be completely embedded in the PA12 matrix. The obtained widths and depths of the HAZ as a measure of uniformity are shown in Figure 16.

For the case without additional heating, a substantial decrease in the width and depth of the HAZ can be seen as a function of the time for roving integration of around $\tau_{HM3}=$117 s. The reason for this can be seen in the strong cooling of the powder bed surface due to radiation and convection losses to the ambient atmosphere of the process chamber. In contrast, the values for width and depth with an additional heat source are more homogenous. It can be seen that there is only a marginal difference in the values between the start and end points. Variations can be explained by the initial cooling, the slow heating of the powder bed surface for longer dwell times, and the reduced heat flow below the nozzle slot. Overall, a more uniform HAZ is created with an additional heat source, laying the basis for more detailed investigations regarding the HAZ formation and roving integration processes.

#### 3.3.2. Evaluation of Process Reliability Using a Flat Pedal for Mountain Bikes

**Figure 17:**
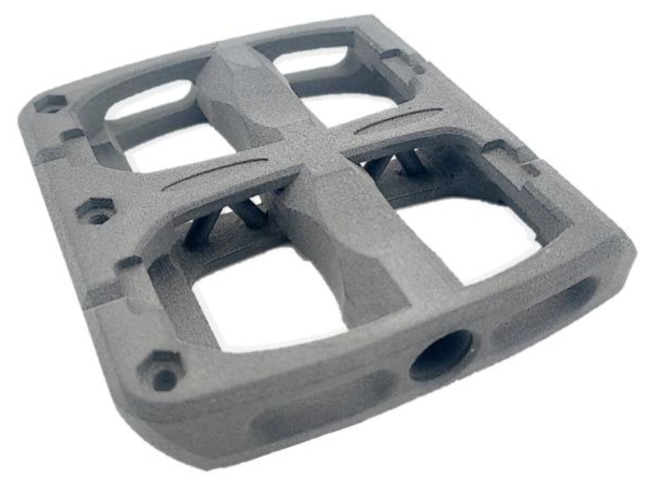
Manufactured flat pedal with 120 integrated rovings.

Figure 17 shows the manufactured flat pedal.

Using this flat pedal, the process reliability for maintaining the sintering window and the reproducible and in-build curling-free integration of 12 rovings in 10 layers each could be demonstrated. Thus, the constancy of the heat distribution during the roving integration was verified. However, the total number of rovings of 120 with an average length of about 75 mm increases the total manufacturing time. For the flat pedal with rovings, an increase in time of approx. 75 min was determined compared to a flat pedal without roving integration. For a time-efficient production of CCFRP parts, the process time for roving integration should be considered and optimised in future investigations.

## 4. Conclusions

Additive manufacturing of CCFRP parts with the LS machine developed aims to combine the process-specific advantages of the LS process with the advantages of continuous fibre reinforcement. With this LS machine, in the future, complex shapes and near-net-shape functional parts with promising basic properties (matrix) will be able to be reinforced in a load-path-oriented manner with the help of 1 K rovings. Thus, CCFRP parts can be produced without support structures and time-consuming post-processing steps. To integrate 1 K rovings into LS parts, the layer-related integration of rovings is carried out with the help of a fibre integration unit. However, this fibre integration unit causes shading of the IR radiation of the IR emitters. The result is a cooling of the part and powder bed surface temperature. The consequence is an uneven HAZ and, thus, an uneven integration depth of the roving in the part layers. If the temperature falls below the crystallisation temperature, the risk of in-build curling increases rapidly. An insufficient integration depth of the roving and in-build curling, thus, forms interfering contours for the recoater. In the worst case, the entire printing process has to be aborted. The consequences are a loss of resources. However, with the help of an additional heat source on the bottom of the fibre integration unit, the part and the powder bed surface temperature are kept within the sintering window during roving integration. Within the scope of this paper, three heat source variants were evaluated regarding the homogeneity of the heat distribution using an IR camera. Based on the best heat source variant, operating points for the air gap width $h_{HM3}$ between the powder bed surface and the temperature of the additional heat source $T_{HM3}$ were identified. These operating points allow a stable and reproducible roving integration within the sintering window in the stable sintering area of the LS machine. A horseshoe part and a flat pedal for mountain bikes with embedded 1 K rovings were used to verify the selected heat source variant and the operating points. In summary, with the help of the selected heat source variant and the operating points found, a process-reliable roving integration can be carried out in the future. The results, thus, form the basis for future investigations. The key conclusions of this work are presented in the following points.

- To keep the powder bed surface reliably warm within the sintering window during roving integration, variant 3 with an aluminium plate with a covered fibre nozzle slot and a glued silicon heating mat with symmetrical surface heat power proved very useful.
- In all heat source variants, there is a temperature drop, especially at the edges and corners, due to the onset of convection flows (Nu > 1). The requirement for adiabatic side walls (validity criteria for Fourier’s law) is, thus, not met in the first two variants, or only to a limited extent (variant 3). Using a steel stiffener mounted on the edges of the additional heat source also favours cooling at the edges.
- A steel plate in variant 2 creates a poorer heat distribution on the powder bed surface, due to the worse thermal conductivity, by a factor of 10 compared to an aluminium plate.
- With the help of a feeler gauge tape and a hold-down device, the fibre nozzle slot in variant 3 is covered, almost entirely preventing the onset of convection. The validity criteria for Fourier’s law can, thus, be better observed. However, the temperature of the metal plate at the point where the hold-down device and the feeler gauge tape are attached is comparatively cool due to the inaccessibility of the silicone heating mat in this area.
- In terms of temperature, reliable operation favoured 175 °C < $T_{HM3}$ ≤ 185 °C for an air gap width of $h_{HM3}$ = 1 mm, and $T_{HM3}$ > 185 °C for an air gap width of $h_{HM3}$ = 1.5 mm for an almost constant power bed surface temperature.
- If the air gap distance $h_{HM3}$ is too small (0.5 mm) and the temperature of the additional heat source $T_{HM3}$ is too high, bulging will occur due to the thermal expansion of the metal plate. The consequence of this bulging is the adhesion or melting of loose powder or the part, thus, a premature abortion of the printing process. If, on the other hand, the air gap distance $h_{HM3}$ and the temperature of the additional heat source $T_{HM3}$ is too low, the initially cold ambient temperature in the process chamber and, thus, in the air gap width $h_{HM3}$ causes a drop in the powder bed surface temperature. The powder bed surface temperature takes some time to heat up. The duration to heat up this enclosed air volume needs more time compared to the air gap widths of 0.5 mm and 1.0 mm.

Optimisation approaches for a more homogeneous heat distribution, and a more controlled retention of the sintering window, are listed in the following points.

- Further development of the additional heat source (variant 3) with a silicone heating mat to be more completely enclosing or a metal plate at the edge areas to avoid convection flows and, thus, an increased temperature decrease in the edge areas.
- Integration of chamber wall heaters into the LS machine to reduce heat losses in the edge areas and to extend the stable build area. More parts can, thus, be printed, reducing the amount of unused powder.
- Reducing the area of the stiffener on the metal plate, so that the silicone heating mat can better heat the edge areas.
- Adaptive temperature control of the silicone heating mat by measuring the temperature of the powder bed surface with an entrained pyrometer. This pyrometer could be placed on a specific spot on the metal plate of the additional heat source.
- Compensate for the initial decrease in temperature of the powder surface by using a higher starting temperature of the heated metal plate, so that the cold air gap can be warmed up as quickly as possible.
- Air gap width control as a function of thermal expansion. However, this point should be analysed in further thermal investigations to confirm the need for air gap control.
- Developing of a wiper concept to remove potential powder build-up on the metal plate and the fibre nozzle. For this purpose, a wire brush could be used to clean the surface.

Future research will focus on further developing of the geometry and optimisation of the edge areas of the heat source. In addition, the target variables of roving integration (shape of the HAZ, process time for roving integration, process reliability) will be optimised with the help of the design of experiments. Determining the mechanical properties as a function of the fibre volume content and the structure–property relationships are also the subject of future investigations.

## Figures and Tables

**Table 1:** Properties of the PA12 powder from Sintratec AG used in this paper [22].

Property	Value
$\mathrm{Emissivity}\;\varepsilon_{p}$	≈0.9
$\mathrm{Melting}\;\mathrm{temperature}\;T_{M,onset}$	≈180 °C
$\mathrm{Crystallisation}\;\mathrm{temperature}\;T_{K,onset}$	≈154 °C
$\mathrm{Sin}\mathrm{tering}\;\mathrm{window}\;∆T_{SW}=T_{M,onset}-T_{K,onset}$	≈26 °C

**Table 2:** Influencing variables for keeping the part/powder bed surface temperature within the sintering window.

Symbol	Description	Unit	Setting	Setting Range
$l_{HM3}$	Length of the metal plate of the additional heat source in the x-direction (see Figure 2b).	mm	laser cutting	≤150
$b_{HM3}$	Width of the metal plate of the additional heat source in the y-direction (see Figure 2b).	mm		<150
$A_{HM3}$	Area of the heat source (metal plate)	mm^2^		≈22,500
$h_{HM3}$	Distance of heat source (metal plate) to powder surface = air gap width	mm	feeler gauge tape	0–2
$\varepsilon_{P}$	Emissivity of the powder (Sintratec PA12)	-	state of delivery	≈0.9
$\varepsilon_{HM3}$	Emissivity of the matt black painted metal plate	-	varnished	≈0.9
$T_{HM3}$	Heat source temperature (metal plate)	°C	PLC	…200
$\tau_{HM3}$	Dwell time	s	PLC	…3600
${\dot{Q}}_{K,B}$	Heat losses due to convection to the surroundings	W	Disturbance variables	
${\dot{Q}}_{S,B}$	Heat losses due to radiation to the environment	W		
${\dot{Q}}_{M}$	Heat losses to the machine structure (especially in the edge area of the building platform)	W		

**Table 3:** Target variables for successful heating of the part/powder bed surface within the sintering window during roving integration.

Symbol	Description	Unit
$T_{O}$	Part/powder bed surface temperature during roving integration. Ideally, $T_{K}<T_{O}<T_{M}.$	°C
∆T	The maximum temperature difference on the powder bed surface between hot spot and cold spot. Ideally, ∆T= 0 °C.	°C
$\tau_{HM3}$	Duration of roving integration / Dwell time of the fibre integration unit over the powder bed surface	s
$b_{HAZ}$	Width of the HAZ	mm
$t_{HAZ}$	Depth of the HAZ	mm

**Table 4:** Technical data of the measuring chain with IR camera.

Technical Data	Description
Lens	Focal length 25 mm
Measuring field size on powder surface	≈140 mm × 110 mm
Camera software	InfraTec IRBIS3
Data evaluation and display	MATLAB
Frame rate	1500 Hz
Adjusted emissivity	0.9
Measurement accuracy	+/−1% of the display value
Temperature resolution at 30 °C	0.015 K
Measuring field size on powder surface	≈140 mm × 110 mm
Isolated optics and laser area	Rock wool

**Table 5:** Process and material parameters of the LS machine selected for the investigations.

Setting	Value
Material	Sintratec PA12 (black)
Mixing ratio	60% Fresh powder/40% Used powder
Number of initial layers in sintering phase	20
Thickness of the powder cake	40 mm
Sintering temperature $T_{O}$	175 °C
Layer thickness	0.1 mm
Inert gas	-
Process chamber temperature	Uncontrollable (≈110 °C)
Heating up time	90 min
Platform heater	170 °C
Standby temperature of the IR emitters during roving integration	170 °C
Rapid traverse of the fibre integration unit in x-direction	3000 mm/min
$\tau_{HM3}$	30 s

**Table 6:** Factor levels to determine the operating points.

Factor	Factor Levels
$T_{HM3}$	175, 180, 185
$h_{HM3}$	0.5, 1, 1.5
$\tau_{HM3}$	15, 30, 60, 90, 120, 150, 180, 210, 240, 270, 300, 330, 360, 390, 420, 450, 480

**Table 7:** Process and material parameters of the developed LS machine.

Setting	Value
Laser spot diameter	≈0.1 mm
Laser output	1.6 W
Hatch distance	0.1 mm
Scan speed	650 mm/s
Layer thickness	0.1 mm
Energy density per unit area $E_{F}$	0.025 J/mm2
Cooling down time	10 h (Overnight)
Platform heater	170 °C
1 K roving	Carbon, 67 tex, HTA40 from Teijin Limited
Coating material	PERICOAT AC250
Coating content	5%
Temperature of fibre nozzle $T_{FN}$	345 °C
Feed rate of fibre nozzle $v_{D}$	120 mm/min
Part thickness at fibre integration	2.5 mm
Distance from fibre nozzle to part bed surface	0.6 mm

## Data Availability

Not applicable.
